# The Pan-Canadian Chemical Library: A Mechanism to Open Academic Chemistry to High-Throughput Virtual Screening

**DOI:** 10.1038/s41597-024-03443-5

**Published:** 2024-06-06

**Authors:** Corentin Bedart, Grace Shimokura, Frederick G. West, Tabitha E. Wood, Robert A. Batey, John J. Irwin, Matthieu Schapira

**Affiliations:** 1 Structural Genomics Consortium, University of Toronto, Toronto, Ontario M5G 1L7 Canada; 2 Univ. Lille, Inserm, CHU Lille, U1286 - INFINITE - Institute for Translational Research in Inflammation, F-59000 Lille, France; 3 Davenport Research Laboratories, Dept. of Chemistry, University of Toronto, 80 St. George Street, Toronto, ON M5S 3H6 Canada; 4 Department of Chemistry, University of Alberta, Edmonton, AB T6G 2G2 Canada; 5 Department of Chemistry, The University of Winnipeg, 515 Portage Avenue, Winnipeg, MB R3B 2E9 Canada; 6 Acceleration Consortium, University of Toronto, Toronto, ON M5S 3H6 Canada; 7 Department of Pharmaceutical Chemistry, University of California San Francisco, San Francisco, California 94143 USA; 8 Department of Pharmacology and Toxicology, University of Toronto, Toronto, Ontario M5S 1A1 Canada

**Keywords:** Databases, Combinatorial libraries

## Abstract

Computationally screening chemical libraries to discover molecules with desired properties is a common technique used in early-stage drug discovery. Recent progress in the field now enables the efficient exploration of billions of molecules within days or hours, but this exploration remains confined within the boundaries of the accessible chemistry space. While the number of commercially available compounds grows rapidly, it remains a limited subset of all druglike small molecules that could be synthesized. Here, we present a workflow where chemical reactions typically developed in academia and unconventional in drug discovery are exploited to dramatically expand the chemistry space accessible to virtual screening. We use this process to generate a first version of the Pan-Canadian Chemical Library, a collection of nearly 150 billion diverse compounds that does not overlap with other ultra-large libraries such as Enamine REAL or SAVI and could be a resource of choice for protein targets where other libraries have failed to deliver bioactive molecules.

## Background & Summary

Strong interest in virtual library screening started to emerge in the 1990s, with the advent of combinatorial chemistry and parallel synthesis^1^. Since then, progress in the field has been incremental, and driven mainly by two factors: the growing size of chemical libraries, and the exponential increase in computational power enabling the screen of ever larger compound collections. Indeed, it is now established that virtually screening larger libraries leads to the discovery of better fitting molecules for a given binding site^2^. A compounding factor is the emergence of deep learning methods that are expected to soon enable robust screening with speed that is not accessible to physics-based approaches^3^.

Based on these observations, sustained efforts are ongoing to increase the size of the synthetically accessible chemical space. The main actors in the field include chemical vendors such as Enamine, WuXi, Otava chemicals, or Mcule, that all have catalogs in the billions of molecules. In particular, Enamine now offers a library of 6 billion make-on-demand compounds from their Enamine REAL database^4^, and 48 billion make-on-demand compounds from the REAL space^5^. A similar quest takes place in industry, where pharmaceutical companies are rapidly growing their searchable chemical space^6^. In the public sector, the Synthetically accessible Virtual Inventory (SaVI) is composed of 1.75 billion compounds accessible with commercial reagents using a collection of 53 chemical reactions^7^.

While the reactions used by chemical vendors and SaVI are generally well known to medicinal chemists, taking advantage of the innovative chemistry invented in academic laboratories could open the gates to vast areas of the chemical space so far less accessible to drug discovery. As a pioneering example, an efficient synthetic scheme for tetrahydropyridines developed by Ellman and colleagues enabled the constitution of a bespoke library of 75 million molecules focused on aminergic G-protein-coupled receptors, and mostly absent from chemical catalogs. Virtual screening of this biased set led to the discovery of the first 5-HT2A receptor agonists with antidepressant activity^8^. Inspired by this approach, we initiated the enumeration of the Pan-Canadian Chemical Library (PCCL), where chemical reactions developed by a growing network of academic chemistry groups across Canada are enumerated into a virtual screening-ready collection of compounds chemically accessible with commercially available reagents and up to two synthetic steps.

The PCCL combines chemical reactions from the academic laboratories of the research groups of Prof. Robert Batey at the University of Toronto, Prof. Tabitha Wood at the University of Winnipeg, and Prof. Frederick West at the University of Alberta. Combined with compatible reagents from the ZINC database, these reactions generate more than 148 billion compounds synthesizable at any cost, and up to 401 million cheap compounds, where “cheap” compounds are defined as made from in stock building blocks listed in the ZINC database with the best combination of price and delivery speed. Among these more affordable molecules, 128 million satisfy Lipinski and Veber druglikeness rules and can be queried and downloaded from the website https://pccl.thesgc.org.

This druglike and inexpensive collection is as diverse as commercial catalogs in terms of physicochemical properties, three-dimensionality, and chemical scaffolds, while its overlap with existing libraries is almost non-existent.

Opening virtual screening to molecules accessible via novel chemistry invented in the public or private sector can explode the boundaries of the accessible chemical space in drug discovery and other fields. The Pan-Canadian Chemical Library showcases the potential of integrating academic ingenuity and *in silico* compound generation to extend the frontiers of chemical exploration. It may also serve as a valuable resource for the development of pharmacological modulators for every human protein by 2035, a goal set by the Target 2035 initiative to explore the unknown biology of the dark proteome and reveal novel opportunities for precision medicine^9,10^.

## Methods

### Chemical reactions

The pilot version of the PCCL was created from six unique chemical reactions. For each reaction, a set of information was requested as part of the workflow:

- Inclusion patterns, determined from the 2D diagram of the chemical reaction in the form of *reagent A + reagent B - > reaction product*, where the reagents are building blocks with specified functional groups involved in the chemical reaction.
- Global exclusion patterns, to exclude functional groups or structures incompatible with the chemical reaction or reaction intermediates, to be applied to all reagents.
- Reagent-specific exclusion patterns, to exclude incompatible functional groups or structures in each reagent, and to describe more precisely what is and is not allowed for each R-group.

Inclusion patterns, exclusion patterns, and the chemical reaction were encoded in SMARTS format, which enables the specification of chemical patterns for each atom or group of atoms. In addition, up to 40 global exclusion rules from ZINC patterns^11^ were added systematically to avoid reactive and unstable functional groups,by first removing from the list the groups corresponding to the chemical reaction studied on a case-by-case analysis.

Finally, for each reaction, up to 100 compounds were selected using a MaxMin algorithm using ECFP-4 2048 bits fingerprints and Tanimoto coefficient to produce a representative collection of 100 reaction products and their respective reagents. The collection was then further visually inspected by chemists who flagged incompatible reagents, leading to additional exclusion filters. After two or three such curation cycles, no chemical outliers were found, and the full library was enumerated.

#### Reactions from the Batey lab, University of Toronto, ON

**Fig. 1:**
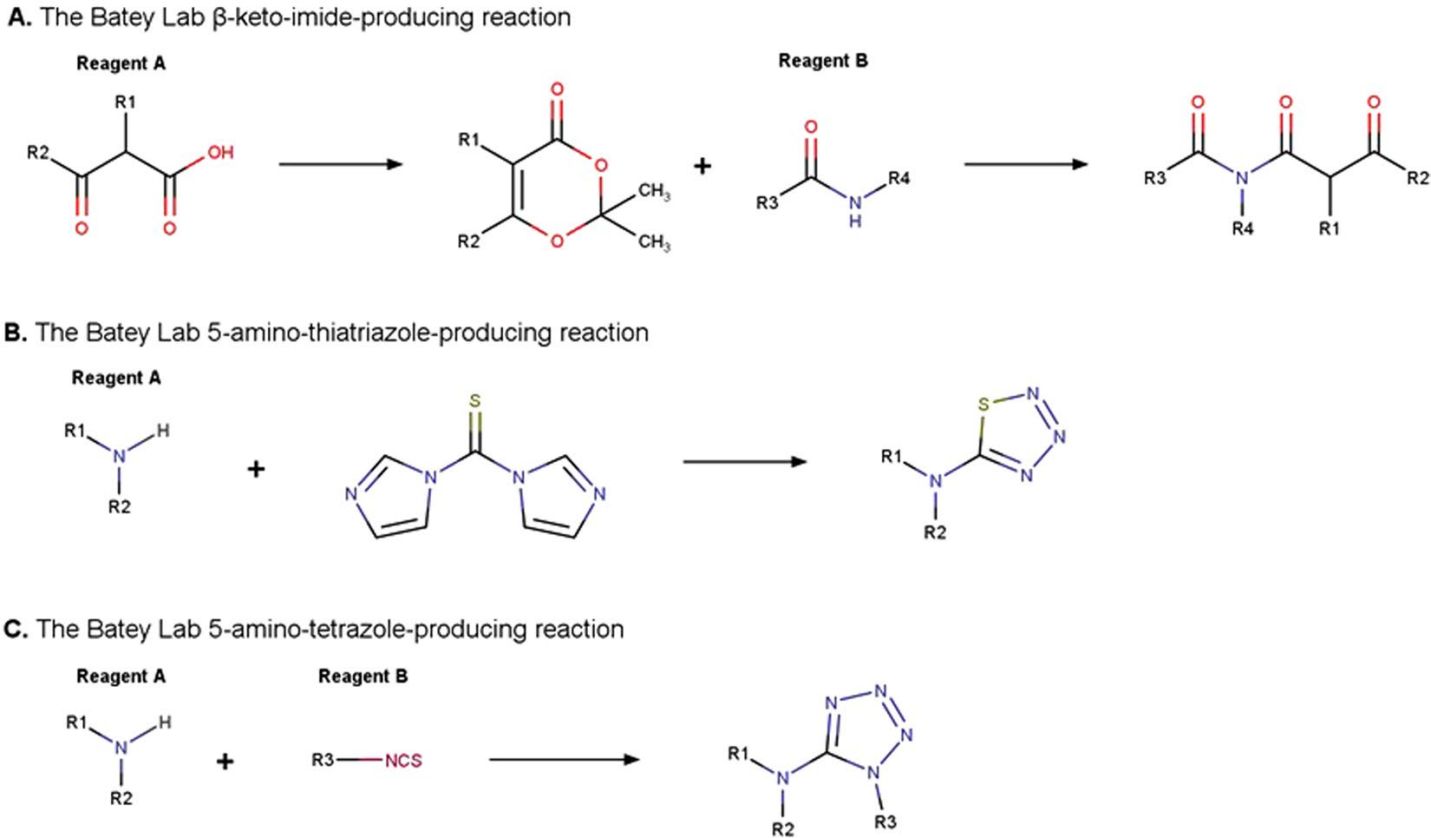
The Batey lab simplified chemical reactions schemes.

Chemical reactions from the Batey lab produced β-keto-imides^12,13^, 5-amino-thiatriazoles^14^, and 5-amino-tetrazoles^15,16^. β-Keto-imide products were enumerated from dioxinones and primary and secondary amides (Fig. 1A, Table 1). Given the low number of dioxinones commercially available, we added an intermediate one-component reaction to obtain them from β-keto acids, including *O*-*tert*-butyl, *O*-methyl, *O*-ethyl and *O*-benzyl protected acidic groups.

**Table 1:** The Batey lab inclusion filters and chemical reactions as SMARTS strings.

**β-keto-imides**	Reagent A precursor	[OH]C([CH2][C;!R]([C;!c])=O)=O
		O=C([CH2][C;!R]([C;!c])=O)OC([CH3])([CH3])[CH3]
		O=C([CH2][C;!R]([C;!c])=O)O[CH3]
		O=C([CH2][C;!R]([C;!c])=O)O[CH2][CH3]
		O=C([CH2][C;!R]([C;!c])=O)O[CH2][c]1[cH1][cH1][cH1][cH1][cH1]1
	Reagent A	C1([CH3])([CH3])OC(=O)C([H])=CO1
	Reagent B	[CX3]([NX3;H2,H1])=O
	Chemical reaction	[O:1]=C1[O:2][C:6](C)(C)[O:3]C([*,H:22])=C1[*,H:21].[*,H:23]C([N:5]([H:99])[*,H:24])=[O:4]⨠[O:4]=C([*,H:23])[N:5]([*,H:24])C(C([*,H:21])C([*,H:22])=[O:3])=[O:1].[H:99][O:2][C:6](C)C
**5-amino-thiatriazoles**	Reagent A	[NX3;H2,H1;!$(NC=O)]
	Chemical reaction	[NX3;H2,H1;!$(NC=O):1]⨠[N:1]C1=NN=NS1
**5-amino-tetrazoles**	Reagent A	[NX3;H2,H1;!$(NC=O)]
	Reagent B	S=C=N[*;!H;!$(C=O)]
	Chemical reaction	[NX3;H2,H1;!$(NC=O):1].S=C=N[*;!H;!$(C=O):3]⨠[*:1](C1=NN=NN1[*:3])

Exclusion filters and reagent mapping for the described chemical reactions are available in the GitHub repository: https://github.com/cbedart/PCCL.

5-Amino-thiatriazoles were enumerated from primary amines, secondary amines, or amino acid derivatives in a one-component chemical reaction (Fig. 1B, Table 1). This reaction included a single variable reagent and led to a small collection of only 7,410 compounds commercially available in the Zinc20 database of 1.4 billion compounds^17^.

5-Amino-tetrazoles were virtually synthesized from primary or secondary amines and isothiocyanates (Fig. 1C, Table 1).

#### Reactions from Wood lab, University of Winnipeg, MB

**Fig. 2:**
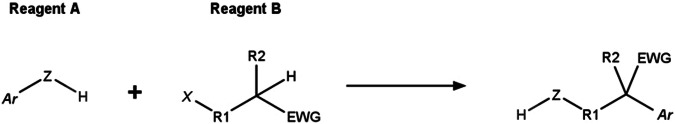
Wood lab simplified chemical reaction scheme.

The reaction submitted by the Wood lab is the Truce-Smiles rearrangement, generating aryl-containing products^18–20^ (Fig. 2, Table 2). In this reaction, the Ar group of reagent A must be any aromatic ring and Z-H either a primary amine, an alcohol, a thiol or a primary sulfonamide group. The R^1^–X group of reagent B represents an acyl halide group (chloride, bromide or iodide), with the carbon ideally positioned within three to five consecutive atoms next to the electron-withdrawing group EWG. Given the configuration of reagents A and B, multiple SMARTS were developed. Reagent A was defined using either the primary amine, alcohol or thiol in the first case, or the primary sulfonamide in the second case. Reagent B was defined by the number of additional carbons between the acyl halide carbon and the central carbon, with 0 to 2 additional sp^3^ carbons bound to 2 hydrogens. In addition, another subdivision was required to differentiate reagents B with R^2^ as a hydrogen atom, leading to non-chiral compounds, from reagents with other R^2^, leading to chiral compounds.

**Table 2:** Wood Research lab inclusion filters and chemical reactions as SMARTS strings.

Reagent A OH/NH_2_/SH	a[OX2H1,NX3H2,SX2H1]
Reagent A SO_2_NH_2_	a[$([SX4](=[OX1])(=[OX1])[NX3H2])]
Reagent B R_2_ = H	O=C([Cl,Br,I])[CX4H2!R,CX4H3!R]([$([CX2]#[NX1]),$([SX4](=[OX1])=[OX1]),$([CX3](=[OX1])),$([CX3](=[OX1])[OX2H0])]) O=C([Cl,Br,I])[CX4H2][CX4H2!R,CX4H3!R]([$([CX2]#[NX1]),$([SX4](=[OX1])=[OX1]),$([CX3](=[OX1])),$([CX3](=[OX1])[OX2H0])]) O=C([Cl,Br,I])[CX4H2][CX4H2][CX4H2!R,CX4H3!R]([$([CX2]#[NX1]),$([SX4](=[OX1])=[OX1]),$([CX3](=[OX1])),$([CX3](=[OX1])[OX2H0])])
Reagent B R_2_ ≠ H	O=C([Cl,Br,I])[CX4H1!R]([$([CX2]#[NX1]),$([SX4](=[OX1])=[OX1]),$([CX3](=[OX1])),$([CX3](=[OX1])[OX2H0])]) O=C([Cl,Br,I])[CX4H2][CX4H1!R]([$([CX2]#[NX1]),$([SX4](=[OX1])=[OX1]),$([CX3](=[OX1])),$([CX3](=[OX1])[OX2H0])]) O=C([Cl,Br,I])[CX4H2][CX4H2][CX4H1!R]([$([CX2]#[NX1]),$([SX4](=[OX1])=[OX1]),$([CX3](=[OX1])),$([CX3](=[OX1])[OX2H0])])
Chemical reactions OH/NH_2_/SH R_2_ = H Non-chiral products	[a:1][OX2H1,NX3H2,SX2H1:2].[F,Cl,Br,I][$(C=O):3][CX4H2!R,CX4H3!R:5][H]⨠[*:5]([a:1])([$(C=O):3][*:2]) [a:1][OX2H1,NX3H2,SX2H1:2].[F,Cl,Br,I][$(C=O):3][CX4H2:6][CX4H2!R,CX4H3!R:5][H]⨠[*:5]([a:1])([*:6][$(C=O):3][*:2]) [a:1][OX2H1,NX3H2,SX2H1:2].[F,Cl,Br,I][$(C=O):3][CX4H2:7][CX4H2:6][CX4H2!R,CX4H3!R:5][H]⨠[*:5]([a:1])([*:7][*:6][$(C=O):3][*:2])
Chemical reactions OH/NH_2_/SH R_2_ ≠ H Chiral products	[a:1][OX2H1,NX3H2,SX2H1:2].[F,Cl,Br,I][$(C=O):3][CX4H1!R:5]([H])([*:9])⨠[*:5]([*:9])([a:1])([$(C=O):3][*:2]) [a:1][OX2H1,NX3H2,SX2H1:2].[F,Cl,Br,I][$(C=O):3][CX4H2:6][CX4H1!R:5]([H])([*:9])⨠[*:5]([*:9])([a:1])([*:6][$(C=O):3][*:2]) [a:1][OX2H1,NX3H2,SX2H1:2].[F,Cl,Br,I][$(C=O):3][CX4H2:7][CX4H2:6][CX4H1!R:5]([H])([*:9])⨠[*:5]([*:9])([a:1])([*:7][*:6][$(C=O):3][*:2])
Chemical reactions SO_2_NH_2_ as non-chiral products	[a:1][$([SX4](=[OX1])(=[OX1])[NX3H2])].[F,Cl,Br,I][$(C=O):3][CX4H2!R,CX4H3!R:5][H]⨠[*:5]([a:1])([$(C=O):3]O) [a:1][$([SX4](=[OX1])(=[OX1])[NX3H2])].[F,Cl,Br,I][$(C=O):3][CX4H2:6][CX4H2!R,CX4H3!R:5][H]⨠[*:5]([a:1])([*:6][$(C=O):3]O) [a:1][$([SX4](=[OX1])(=[OX1])[NX3H2])].[F,Cl,Br,I][$(C=O):3][CX4H2:7][CX4H2:6][CX4H2!R,CX4H3!R:5][H]⨠[*:5]([a:1])([*:7][*:6][$(C=O):3]O)
Chemical reactions SO_2_NH_2_ as chiral products	[a:1][$([SX4](=[OX1])(=[OX1])[NX3H2])].[F,Cl,Br,I][$(C=O):3][CX4H1!R:5]([H])([*:9])⨠[*:5]([*:9])([a:1])([$(C=O):3]O) [a:1][$([SX4](=[OX1])(=[OX1])[NX3H2])].[F,Cl,Br,I][$(C=O):3][CX4H2:6][CX4H1!R:5]([H])([*:9])⨠[*:5]([*:9])([a:1])([*:6][$(C=O):3]O) [a:1][$([SX4](=[OX1])(=[OX1])[NX3H2])].[F,Cl,Br,I][$(C=O):3][CX4H2:7][CX4H2:6][CX4H1!R:5]([H])([*:9])⨠[*:5]([*:9])([a:1])([*:7][*:6][$(C=O):3]O)

Exclusion filters and reagent mapping for the described chemical reactions are available in the GitHub repository: https://github.com/cbedart/PCCL.

The specificity of the Truce-Smiles rearrangement is the inversion of the R^1^ group with the additional carbons in the final product^19^. As it was not possible to create a single SMARTS for all types of R^1^, 12 chemical reactions coded in SMARTS format had to be created based on the 2 different conditions for reagent A and 6 different conditions for reagent B.

#### Reactions from the West lab, University of Alberta, AB

**Fig. 3:**
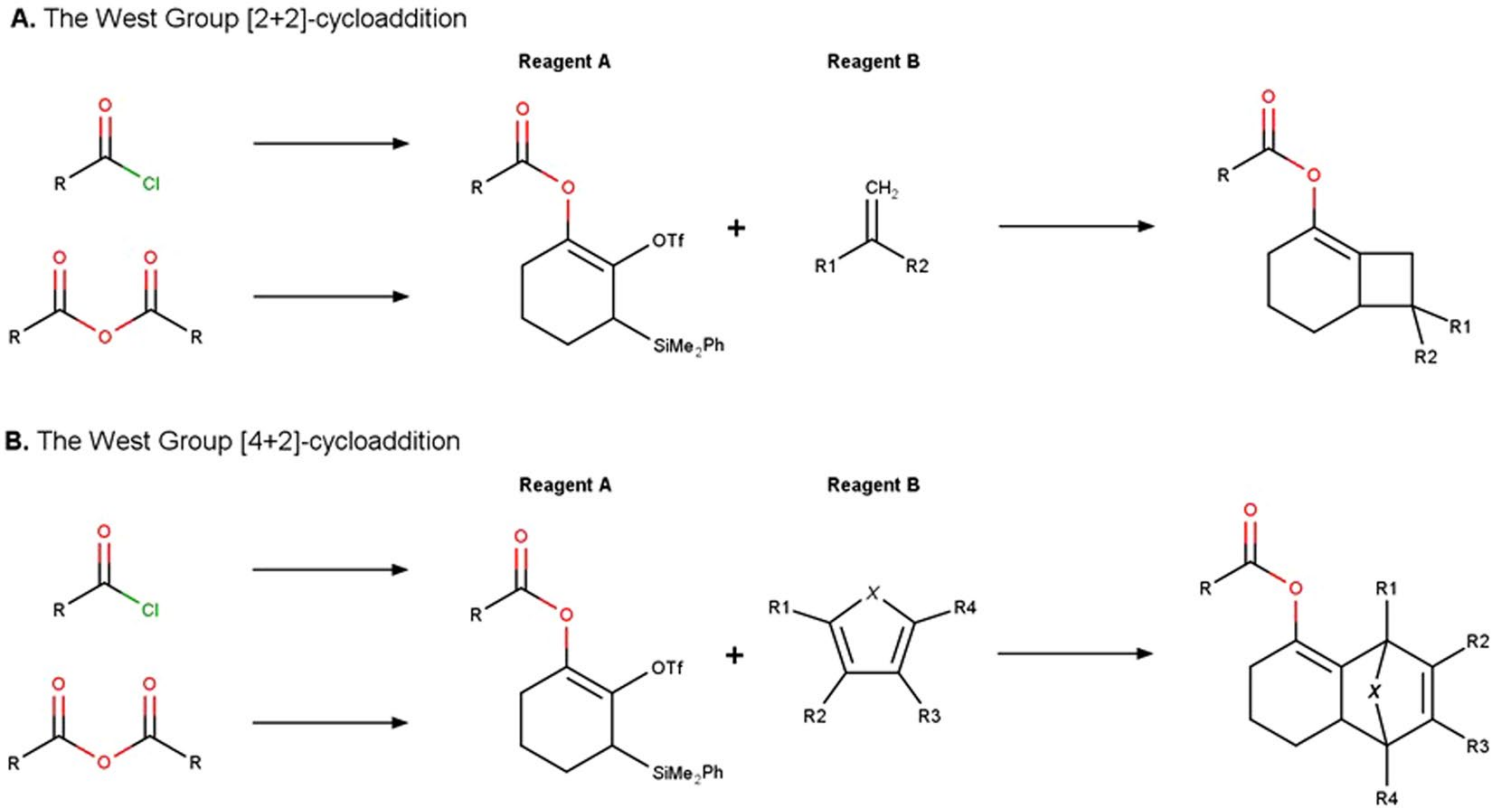
The Westgroup simplified chemical reaction schemes.

The reactions proposed by the West lab are [2 + 2]- and [4 + 2]-cycloadditions, generating bicyclooctenes and bridged tricyclic products via the generation of cyclic allenes^21–24^ (Fig. 3, Table 3). These reactions require the same reagent A: 1,2-acyloxycyclohexadienes. However, as this family of compounds is not commercially available in sufficient diversity, it is necessary to synthesize them upstream from anhydrides or acyl chlorides^24^.

**Table 3:** The West group inclusion filters and chemical reactions as SMARTS strings.

Reagent A Anhydride		[#6][CX3D3!R](=O)[O!R][CX3D3!R](=O)[#6]
Reagent A Acyl chloride		[#6][CX3D3!R](=O)Cl
		Symmetric reagents defined as [*:1]C(=O)OC(=O)[*:2]
**[2 + 2]-cycloaddition**	Reagent B R_2_ = H	[CX3H2;!R]=[CX3H1;!R]([$([CX3](=[OX1])[#6,$([OX2][#6])]),$([CX2]#[NX1]),c])
	Reagent B	[CX3H2;!R]=[CX3;!R]([#6])([$([CX3](=[OX1])[#6,$([OX2][#6])]),$([CX2]#[NX1]),c])
	R_2_ ≠ H	
	Chemical reactions	C(=O)(OC=O)[*:1].[CX3H2]=C([*:2])([*:3])⨠C1(=C2C(CCC1)C(C2)([*:2])[*:3])OC([*:1])=O
		ClC(=O)[*:1].[CX3H2]=C([*:2])([*:3])⨠C1(=C2C(CCC1)C(C2)([*:2])[*:3])OC([*:1])=O
		C(=O)(OC=O)[*:1].[CX3H2]=C([*:2])([*:3])⨠C1(=C2C(CCC1)C(C2)([*:2])[*:3])OC([*:1])=O
		ClC(=O)[*:1].[CX3H2]=C([*:2])([*:3])⨠C1(=C2C(CCC1)C(C2)([*:2])[*:3])OC([*:1])=O
**[4 + 2]-cycloaddition**	Reagent B Furan	[cX3H1]1o[cX3H1][cX3H0&$(c!@[#6]),cX3H1][cX3H0&$(c!@[#6]),cX3H1]1
		[cX3H0&$(c!@[#6])]1o[cX3H1][cX3H0&$(c!@[#6]),cX3H1][cX3H0&$(c!@[#6]),cX3H1]1
		[cX3H0&$(c!@[#6])]1o[cX3H0&$(c!@[#6])][cX3H0&$(c!@[#6]),cX3H1][cX3H0&$(c!@[#6]),cX3H1]1
	Reagent B Cyclopentadiene	[CX3H1]1=[CX3H0&$(C!@[#6]),CX3H1][CX3H0&$(C!@[#6]),CX3H1]=[CX3H1][CX4H2]1
		[CX3H0&$(C!@[#6])]1=[CX3H0&$(C!@[#6]),CX3H1][CX3H0&$(C!@[#6]),CX3H1]=[CX3H1][CX4H2]1
		[CX3H0&$(C!@[#6])]1=[CX3H0&$(C!@[#6]),CX3H1][CX3H0&$(C!@[#6]),CX3H1]=[CX3H0&$(C!@[#6])][CX4H2]1
	Reagent B Pyrrole	[cX3H1]1n([#6!c])[cX3H1][cX3H0&$(c!@[#6]),cX3H1][cX3H0&$(c!@[#6]),cX3H1]1
		[cX3H0&$(c!@[#6])]1n([#6!c])[cX3H1][cX3H0&$(c!@[#6]),cX3H1][cX3H0&$(c!@[#6]),cX3H1]1
		[cX3H0&$(c!@[#6])]1n([#6!c])[cX3H0&$(c!@[#6])][cX3H0&$(c!@[#6]),cX3H1][cX3H0&$(c!@[#6]),cX3H1]1
		Chemical reactions C(=O)(OC=O)[*:1].[c:2]1[o:3][c:4][c:5][c:6]1⨠C1(=C2C(CCC1)[C:2]3[O:3][C:4]2[C:5]=[C:6]3)OC(=O)[*:1]
		ClC(=O)[*:1].[c:2]1[o:3][c:4][c:5][c:6]1⨠C1(=C2C(CCC1)[C:2]3[O:3][C:4]2[C:5]=[C:6]3)OC(=O)[*:1]
		C(=O)(OC=O)[*:1].[C:2]=1[C:3][C:4]=[C:5][C:6]1⨠C1(=C2C(CCC1)[C:2]3[C:3][C:4]2[C:5]=[C:6]3)OC(=O)[*:1]
		ClC(=O)[*:1].[C:2]=1[C:3][C:4]=[C:5][C:6]1⨠C1(=C2C(CCC1)[C:2]3[C:3][C:4]2[C:5]=[C:6]3)OC(=O)[*:1]
		C(=O)(OC=O)[*:1].[c:2]1[n:3][c:4][c:5][c:6]1⨠C1(=C2C(CCC1)[C:2]3[N:3][C:4]2[C:5]=[C:6]3)OC(=O)[*:1]
		ClC(=O)[*:1].[c:2]1[n:3][c:4][c:5][c:6]1⨠C1(=C2C(CCC1)[C:2]3[N:3][C:4]2[C:5]=[C:6]3)OC(=O)[*:1]

Exclusion filters and reagent mapping for the described chemical reactions are available in the GitHub repository: https://github.com/cbedart/PCCL.

In the case of the [2 + 2]-cycloaddition, reagent B is a styrene or an electron-deficient olefin (Fig. 3A). To consider all possible cases, reagent B was separated into two categories, whether it contains one (1-substituted with R^2^ as H) or two (1,1-substituted with R^2^ ≠ H) substituents.

The case of the [4 + 2]-cycloaddition is more complex, as several families of reagent B can be accepted depending on the type of the atom X in the 5-membered ring (Fig. 3B). Reagent B can be either a furan, a cyclopentadiene, or a pyrrole, where X is an oxygen, carbon or nitrogen-based substituent respectively. In addition, some reagents may be incompatible if they are too sterically hindered in positions R^1^ and R^3^. To provide several sets of enumerated compounds according to their hindrance, all families of reagents B were divided into three categories, where R^1^ and R^3^ are both hydrogens atoms, R^1^ or R^3^ is a hydrogen atom, and neither is a hydrogen.

As a result of the many variations in reagents A and B, there are a total of 4 chemical reactions encoded into SMARTS strings for the [2 + 2]-cycloaddition, and a total of 6 for the [4 + 2]-cycloaddition.

### Building blocks

We searched the Zinc database on the Arthor website (arthorbb.docking.org) to identify compatible building blocks for each chemical reaction^17^. This database, updated in the first quarter of 2022, categorizes commercial building blocks based on their availability and price across five groups^25^.

- The BB-50 group includes in-stock building blocks with the best combination of price and delivery speed.
- The BB-40 group includes second tier in-stock building blocks.
- The BB-30 group includes in-stock building blocks with information that cannot be accurately verified.
- The BB-20 group includes make-on-demand building blocks, with delivery around 6 weeks and a price above 500 USD per 100 mg.
- The BB-10 group includes make-on demand building blocks with delivery around 6 weeks and a price above 1000 USD per 100 mg, as well as expensive in-stock building blocks.

To facilitate the process, we organized these different groups into different categories, “cheap” and “expensive”. The cheap category includes affordable in-stock building blocks from groups BB-50 and BB-40. The expensive category includes all other affordable in stock compounds, make-on-demand and expensive in-stock building blocks from groups BB-30, BB-20, and BB-10.

Building blocks downloaded from Arthor were then subjected to exclusion filters using RDKit^26^. In addition, building blocks were limited in size to 40 heavy atoms. All reagents were saved in SMILES format.

#### Enumeration and physicochemical descriptors

The 2D enumeration of the chemical libraries was performed using python3 scripts based on RDKit. With the help of python3 multiprocessing library, this step was executed on a large-scale using computing resources from the Digital Research Alliance of Canada (DRAC). All reagent SMILES files were divided into groups of up to 2,000 building blocks, to divide the enumeration into 48 or 64 CPU threads depending on the DRAC cluster used. Physicochemical parameters were generated using the QED module^27^. Structural alerts were processed using the RDKit FilterCatalog module. In this study, we applied the Pan assay interference patterns PAINS, separated into three sets PAINS A, PAINS B and PAINS C, to identify compounds that can interact non-specifically and give false positive results^28^, the Brenk filters to flag unwanted functionality due to potential tox reasons or unfavorable pharmacokinetics^29^, and the NIH filters to annotate compounds with reactive or undesired functional groups as well as fluorescent compounds^30,31^.

Some of the physicochemical parameters were used to apply drug likeness rules, including Lipinski’s rule of five^32^ and Veber’s rule^33^. The output included all the parameters used to define the druglike subset, Fsp3, QED^27^, structural alerts, InChiKey and reagents identifiers.

Additional modules were developed to provide information on Bemis-Murcko scaffolds to assess scaffold and structural diversity^34^, principal moments of inertia with the normalized ratio NPR1 and NPR2 to assess the shape of the compounds^35^, and the partitioning of InChiKeys into several files for chemical identity searches with other databases. The principal moments of inertia were performed based on the method described by Irwin *et al*.^11^. Using RDKit, the distance-geometry-based conformer generator EmbedMolecule was used to quickly obtain three-dimensional conformations, and the rdMolDescriptors module generated the NPR1 and NPR2 parameters. The data was then binned using pandas and numpy libraries in 200 × 200 bins for better data management and graph observation. The Bemis-Murcko scaffolds were generated using the *MurckoScaffold.GetScaffoldForMol* function from RDKit. Statistical analysis and overlap between different libraries were performed using the pandas library^36^. Finally, an InChiKey partitioning was generated, by registering the InChiKeys in different directories and files based on their number of heavy atoms and the first two letters of the InChiKey. The presence or absence of the compound in another library was then verified using the bash function grep from a python3 script running in parallel on up to 64 CPU threads.

#### PostgreSQL/RDKit data management and website development

All cheap and druglike compounds from the PCCL were enumerated and stored in a PostgreSQL database with native RDKit cartridge implementation. From the import of a molecule in SMILES format, a PostgreSQL database can efficiently generate a wide range of molecular descriptors, manage substructure and similarity searches from fingerprints also calculated by the database, or generate 2D pictures in a SVG format.

Cheap druglike PCCL compounds were imported in the database from a list in CSV format including the SMILES string, the identifier given by the compound during the enumeration, the physicochemical parameters generated to filter the druglikeness of the compounds, the calculated Fsp3 and QED, and the ZINC identifiers of reagents. For greater practicality and scalability, each chemical reaction was separated into distinct tables.

A website available at https://pccl.thesgc.org/ was developed using a combination of HTML, JavaScript and PHP to make the cheap and druglike compounds database accessible to the scientific community. Users can visualize and download in smiles format any list of compounds satisfying their specified structural queries (drawn with the javascript applet JSME Molecule Editor^37^), physicochemical or QED descriptor restrictions. Descriptors statistics and plots for all chemical reactions are also made available using the JavaScript charting library Chart.js^38^.

## Data Records

The 127.5 million compounds of the Pan-Canadian Chemical Library, composed of druglike compounds affordable to synthesize, can be explored at https://pccl.thesgc.org/, and can be downloaded from Zenodo at https://zenodo.org/records/11371919^39^. The PCCL library hosted on Zenodo is split by reaction, then by number of heavy atoms. Two types of files are available in zip archives:

- The SMILES format files (delimited by a tab character), with the SMILES string and their product name.
- The CSV format file (delimited by comma characters), with all the information generated during their enumeration: ZINC ID of reagents, druglike properties, and purchasability.

The purchasability value is defined by two integers:

- 1 for products only composed of BB-50 reagents.
- 2 for products composed of at least one BB-40 reagent, in combination with one BB-50 or one BB-40.

Detailed inclusion and exclusion filters, as well as the encoded chemical reactions, are all available in the GitHub repository https://github.com/cbedart/PCCL in the “PCCL_reactions” section. For each chemical reaction, two types of files are available.

The “reagents” text files, with the names formatted as “REACTION_Reagents.txt”, containing:

- The synthon SMARTS with an associated Synthon ID for each type of reagent used.
- The symmetric synthon SMARTS filter in the case of symmetrical reagents.
- Synthon-specific exclusion SMARTS filters for each Synthon ID.
- Reaction tags for each Synthon ID.

The “reactions” text files, with the names formatted as “REACTION_Reactions.txt”, containing the reaction SMARTS, an associated Reaction ID, and the mapping of the chemical reactions using the reaction tags for each Synthon ID defined in the “REACTION_Reagents.txt” file.

Based on the information provided, all the 148 billion compounds can be enumerated.

## Technical Validation

### Composition of the database

The construction of the pilot version of the Pan-Canadian Chemical Library was initially focused on β-keto-imides, 5-amino-thiatriazoles, and 5-amino-tetrazoles, Truce-Smiles reaction products, bicyclooctenes, and bridged tricyclics. The library enumeration was based on the ZINC building blocks via the Arthor database, where a total of 165.2 million compatible building blocks with a maximum of 40 heavy atoms were identified, including 1.9 million low-cost compounds. Following the use of the exclusion rules defined above, reagents not compatible with each chemical reaction were removed, resulting in a total of 76.8 million compatible building blocks, 736,639 of which were low-cost (Table 4). Building block availability was highly variable across all chemical reactions, ranging from 305 reagent Bs for Truce-Smiles reactions to 40,091,545 reagent As for 5-amino-thiatriazoles.

**Table 4:** Number of commercially available building blocks for each chemical reaction from Arthor database, after filtering with exclusion filters.

		Cheap reagents	All reagents
β-keto-imides	A	197	7,711
	B	134,392	3,008,552
5-amino-thiatriazoles	A	210,947	40,091,545
5-amino-tetrazoles	A	185,861	26,076,455
	B	792	3,489
Truce-Smiles	A	144,346	6,109,705
	B	39	305
[2 + 2]-cycloaddition	A	4,313	21,756
	B	13,387	76,539
[4 + 2]-cycloaddition	A	4,313	21,756
	B	38,052	1,41,2588

Using commercially available building blocks, a total of 148 billion compounds were enumerated, including 401 million cheap compounds (Table 5).

**Table 5:** Number of enumerable compounds for each chemical reaction.

	Cheap products	All products
β-keto-imides	26,475,224	23,198,944,472
5-amino-thiatriazoles	72,045	36,330,568
5-amino-tetrazoles	147,201,912	90,980,751,495
Truce-Smiles	5,629,494	1,863,460,025
[2 + 2]-cycloaddition	57,738,131	1,665,182,484
[4 + 2]-cycloaddition	164,118,276	30,732,264,528
**Total**	401,235,082	14,847,6933,572

### Enumeration of Cheap/Druglike subsets

**Fig. 4:**
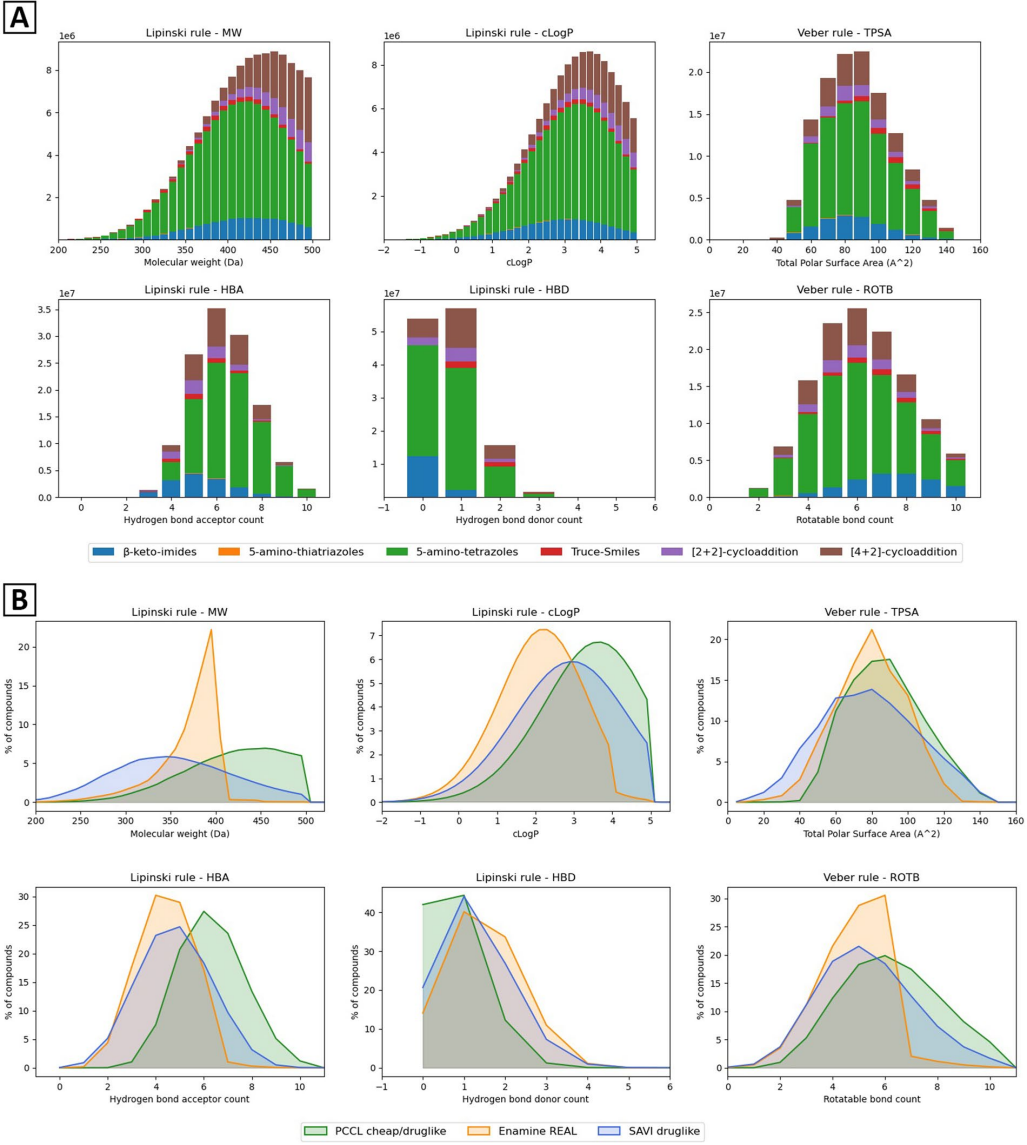
(**A**) Distribution of physicochemical descriptors for each enumerated library (**B**) Main physicochemical parameters distribution for the druglike subset of the PCCL (in green), Enamine REAL (in orange), and druglike-filtered SAVI 2020 (in blue) databases.

A druglike library of 127.5 million compounds accessible with cheap reagents was compiled using the Lipinski and Veber rules described above, stored in a postgreSQL/RDKit database, and made available on https://pccl.thesgc.org (Table 6). The distribution in physicochemical descriptors varies depending on the chemical reaction used to enumerate the library (Fig. 4A). In particular, [2 + 2]- and [4 + 2]-cycloadditions produce larger compounds due to the large core scaffolds created during the reactions. At the opposite end of the molecular weight spectrum, 5-amino-thiatriazoles are smaller as they involve a single building block.

**Table 6:** Number of cheap and druglike compounds for each chemical reaction.

	Cheap products	Cheap & druglike products
β-keto-imides	26475,224	14,255,715
5-amino-thiatriazoles	72,045	198,795
5-amino-tetrazoles	147,201,912	80,424,292
Truce-Smiles	5,629,494	3,431,098
[2 + 2]-cycloaddition	57,738,131	7,177,863
[4 + 2]-cycloaddition	164,118,276	22,050,523
**Total**	401,235,082	127,538,286

### Comparison with enamine REAL and SAVI databases

The main goal of the PCCL is to open new chemical spaces not covered by existing chemical libraries for applications in chemical biology, drug discovery or other fields. To evaluate its chemical diversity, we compared this first version of the PCCL with two ultra large commercial and academic libraries, Enamine REAL and the Synthetically Accessible Virtual Inventory (SAVI) respectively (Table 7). We used the June 2023 version of Enamine REAL containing 6 billion druglike molecules and the April 2020 version of SAVI, a library developed by the NIH National Cancer Institute, with 1.75 billion compounds. Since not all SAVI compounds were druglike, we filtered the library with the same scripts and rules used to create the druglike subset of the PCCL, leading to a SAVI library of 1.4 billion molecules.

**Table 7:** Data for Enamine REAL and SAVI 2020 databases compared to the cheap and druglike subset of the PCCL.

		PCCL	Enamine REAL - 2023	SAVI - 2020
	Compounds	128 207 251	6 039 411 638	1 417 282 927
	Subset	Cheap & Druglike	Druglike	Druglike filtered
	Price	Cheap	Cheap/“Advanced”	“Enamine in stock building blocks”
	Latest update	August 2023	June 2023	April 2020
Lipinski & Veber rules	HAC	Up to 38	Up to 38	Up to 38
	MW	≤500 Da	≤500 Da	≤500 Da
	LogP	≤5	≤5	≤5
	HBA	≤10	≤10	≤10
	HBD	≤5	≤5	≤5
	ROTB	≤10	≤10	≤10
	TPSA	≤140	≤140	≤140
Filters	PAINS	2.55%	0.29%	2.77%
	BRENK	43.68%	24.9%	34.16%
	NIH	13.41%	3.84%	9.75%

Using RDKit filter catalogs, we evaluated the proportion of compounds flagged as problematic in each chemical library. The percentage of compounds flagged by the various filters was similar in the PCCL, while Enamine REAL fared better on the various structural alerts. For instance, 2.55% of PCCL compounds and 2.77% of SAVI compounds were flagged as PAINS, compared with 0.29% of Enamine REAL compounds (Table 7 - Filters).

### Physicochemical statistics

Using the same methods as above, we compared the distribution of the main physicochemical descriptors across the different libraries. A significant difference in terms of molecular weight distribution between Enamine REAL, SAVI, and the PCCL was observed. Enamine REAL seems to offer a large majority of compounds with a molecular weight below 400 Da, that can be functionalized in hit-to-lead processes while remaining within the limits of Lipinski’s rule of five. The filtered SAVI library also features a majority of compounds below 400 Da. By analyzing the building blocks used by SAVI on their website, this distribution is achieved through the use of small building blocks, with an average weight of 212 Da and 13.5 heavy atoms^40^. While still satisfying Lipinski and Veber rules, compounds from the PCCL are larger and synthesized from building blocks with an average weight between 230 and 290 Da, and an average heavy atom count between 16 and 20, depending on the chemical reactions (Fig. 4B). Molecules with lower molecular weight are typically better chemical starting points for lead optimization, but larger compounds may be necessary to generate hits for challenging proteins with shallow binding sites. Importantly, the number of hydrogen-bond donors in PCCL compounds remains low, a necessity as, unlike other Lipinski boundaries, a maximum of five hydrogen bond donors is a limit that cannot be transgressed^41^.

### Three-dimensional properties

**Fig. 5:**
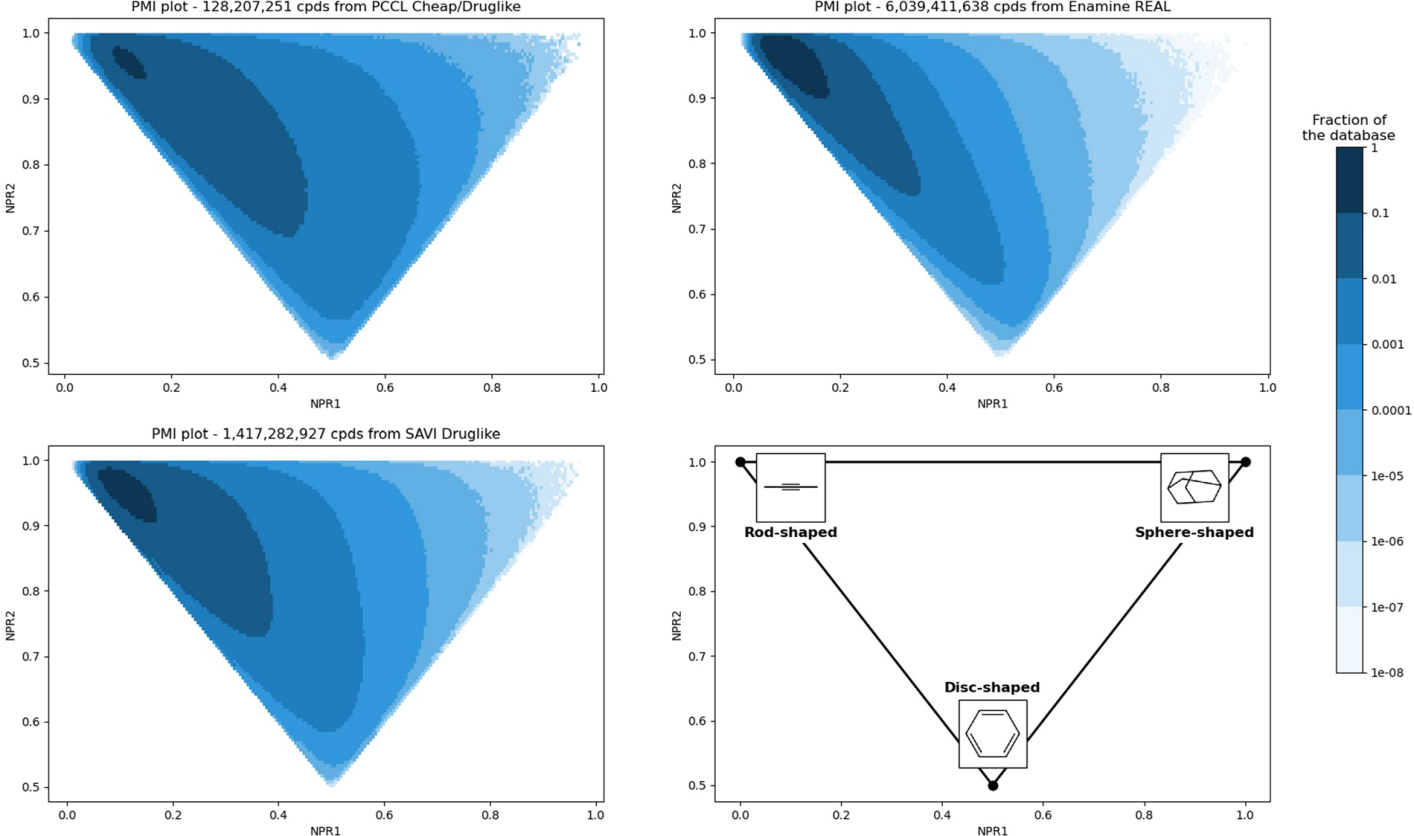
Molecular shape distribution of PCCL, Enamine REAL, and druglike-filtered SAVI, from a Principal Moments of Inertia analysis leading to the calculation of normalized PMI ratios NPR1 and NPR2. A pixel corresponds to a specific percentage of the database defined by its color.

The three-dimensional shapes of every chemical library were analyzed using the normalized principal moments of inertia (PMI) ratios NPR1 and NPR2^35^, leading to 2D plots of chemical libraries where the top-left corner represents one-dimensional rod-like molecules, the bottom is populated with planar compounds and the top-right corner is filled with three-dimensional molecules (Fig. 5). The PCCL covers the same disc-shaped and rod-shaped areas, at the top left corner of the PMI triangle. The main benefit of the PCCL library compared to Enamine REAL is the proportionally different coverage of highly three-dimensional spaces, historically underrepresented, to the sphere-shaped area at the top right corner.

### Chemical diversity and novelty

To assess the chemical diversity of the cheap and druglike PCCL, its Bemis-Murcko Scaffolds composition^34^ was compared to that of other libraries (Table 8). We found that the PCCL and SAVI druglike collections had on average 14 compounds per Bemis-Murcko scaffold, compared with 17 for Enamine REAL, reflecting a modest increase of 20% in the diversity of the PCCL and SAVI libraries. This difference correlates with the average number of compounds produced per reaction, which is 21.4 million for PCCL and 36.2 million for Enamine REAL. This also indicates that on average, a slightly wider range of analogs should be available for any given hit compound from Enamine REAL. But we envision that if a hit is identified from the cheap PCCL, analogs could also be sought after from the much larger set of >150 billion less affordable PCCL compounds. The Bemis-Murcko Scaffold composition of this collection was not analyzed due to its overwhelming size, but since it is generated from the same set of six chemical reactions, we expect that it would include a wide range of analogs for any molecule from the cheap and druglike PCCL set.

**Table 8:** Bemis-Murcko Scaffolds for the druglike subset of the PCCL, Enamine REAL, and druglike-filtered SAVI 2020 databases.

	Compounds	Bemis-Murcko Scaffolds	Compounds per scaffold
PCCL cheap/druglike	128 207 251	9 244 232	13.87
Enamine REAL	6 039 411 638	354 179 312	17.05
SAVI druglike	1 417 282 927	99 955 822	14.18

The chemical novelty of the PCCL was first assessed by calculating the overlap of its Bemis-Murcko scaffolds with the other libraries (Table 9). The overlap in chemical scaffolds is clearly negligible: 0.29% of scaffolds found in the cheap and druglike PCCL are also found in Enamine REAL, and 0.25% in the druglike SAVI collection. This in contrast with a significant overlap between the other two libraries, where 21.57% of SAVI scaffolds are also found in Enamine REAL.

**Table 9:** Comparison of the number of Bemis-Murcko Scaffolds shared between two chemical libraries.

	Bemis-Murcko Scaffolds	Shared with PCCL		Shared with REAL		Shared with SAVI	
PCCL cheap/druglike	9 244 232			26 835	0.29%	23 265	0.25%
Enamine REAL	354 179 312	26 835	0.007%			21 559 452	6.09%
SAVI druglike	99 955 822	23 265	0.02%	21 559 452	21.57%		

To confirm the chemical novelty of the PCCL, we used InChiKey representations of the molecules to determine the presence or absence of each fully enumerated cheap and druglike PCCL compound in the Enamine Real and druglike SAVI collections (Table 10). This analysis reinforced the previous results: only 21,581 out of 128,207,251 PCCL compounds can be found in Enamine REAL, and only 33,050 in SAVI, representing an overlap below 0.03% in both cases. Limitations in computing power precluded us from comparing the SAVI set with the 6 billion REAL compounds, but we were able to conduct the analysis with the 2020 version of Enamine REAL containing 1.2 billion compounds. Here, we found 142.8 million identical molecules, representing an overlap of 11.9% between Enamine REAL and SAVI libraries. This probably reflects the fact that numerous chemical reactions used to generate SAVI are underlying the Enamine collection, such as Hartenfeller’s collection of chemical reactions^42^. Together, these results confirm that a library such as the PCCL, derived from chemical reactions that are underexplored in medicinal chemistry, opens-up a novel and diverse chemical space for drug discovery.

**Table 10:** Comparison of the compounds shared between two chemical libraries.

	Identity with Enamine REAL	Identity with SAVI druglike
PCCL cheap/druglike	21 581	33 050
	0.017%	0.026%

### Synthesis success rate

The average success rate for the chemical synthesis of PCCL compounds is not well defined. We anticipate that in some cases (such as reactions from the Batey lab above), it is close to the ~80% success rate provided by commercial vendors^43,44^, but we expect that it will vary from one reaction to another. A mechanism that may be implemented in the future would be to synthesize 50 or more representative compounds to experimentally evaluate synthesis success rate before any new reaction is added to the PCCL.

## Usage Notes

We envision that the primary use of the PCCL is the discovery of hit molecules for challenging target classes where other libraries have failed to deliver a chemically tractable hit. As more chemical reactions underexplored in medicinal chemistry are incorporated, we expect that the PCCL will grow in the trillions of molecules. The more limited cheap and druglike collection will probably reach billions of compounds. Given the low experimental confirmation rate of computational hit candidates, we anticipate that primary virtual screening will focus on this smaller, more affordable set, while hit expansion could benefit from the full PCCL collection.

Even with relatively modest computing resources, modern AI-accelerated or synthon-based virtual screening techniques (where the synthons rather than the combinatorially enumerated library are screened and then assembled) are well adapted to screen such ultra-large libraries. One example is the hierarchical structure-based screening, introduced by Zhou *et al*. in 2009^45^, and made popular by the V-SYNTHES software developed by Sadybekov *et al*. in 2021^46^. To facilitate the application of synthon-based screening to the PCCL, we developed SATELLiTES (Synthon-based Approach for the Targeted Enumeration of Ligand Libraries and Expeditious Screening), a freely available software available at https://github.com/cbedart/SATELLiTES that requires chemical reactions in SMARTS format as input and generates virtual-screening-ready collections of commercially available synthons where the reactive functional group is replaced by a simple chemotype of choice, such as a methyl group (to be published). Synthon hit candidates are then automatically combined by SATELLiTES into small collections of fully enumerated molecules for rapid virtual screening.

We hope that the PCCL will prove a successful and convincing paradigm where chemical reactions developed in academia or the industry that are typically overlooked in large commercial libraries are used to open uncharted areas of the chemical space for virtual screening, with potential applications in drug discovery, material sciences and other fields. While our choice to focus here on Canadian chemistry groups is meant to facilitate operations and driven by the nationally fragmented nature of funding mechanisms in academia, the process could in principle be expanded across borders. Ideally, future breakthroughs in computational hit prediction, maybe driven by artificial intelligence and revealed by benchmarking challenges such as CACHE^47^, will turn this novel library screening paradigm into a well-established modus operandi.

## Data Availability

The source code of the Pan-Canadian Chemical Library website is available in the GitHub repository https://github.com/cbedart/PCCL in the “PCCL_website” section. All Python scripts used to generate the Pan-Canadian Chemical Library are to be compiled into a single Python package named the Bespoke Library Toolkit (BLT): https://github.com/cbedart/BespokeLibraryToolkit and are available upon request from MS.

## References

[1] Bunin BA, Plunkett MJ, Ellman JA (1996). Synthesis and evaluation of 1,4-benzodiazepine libraries. Methods Enzymol..

[2] Lyu J, Irwin JJ, Shoichet BK (2023). Modeling the expansion of virtual screening libraries. Nat. Chem. Biol..

[3] Kimber TB, Chen Y, Volkamer A (2021). Deep Learning in Virtual Screening: Recent Applications and Developments. Int. J. Mol. Sci..

[4] REAL Database - Enamine. https://enamine.net/compound-collections/real-compounds/real-database.

[5] REAL Space - Enamine. https://enamine.net/compound-collections/real-compounds/real-space-navigator.

[6] Warr WA, Nicklaus MC, Nicolaou CA, Rarey M (2022). Exploration of Ultralarge Compound Collections for Drug Discovery. J. Chem. Inf. Model..

[7] Patel H (2020). SAVI, in silico generation of billions of easily synthesizable compounds through expert-system type rules. Sci. Data.

[8] Kaplan AL (2022). Bespoke library docking for 5-HT2A receptor agonists with antidepressant activity. Nature.

[9] Carter AJ (2019). Target 2035: probing the human proteome. Drug Discov. Today.

[10] Müller S (2022). Target 2035 – update on the quest for a probe for every protein. RSC Med. Chem..

[11] ZINC20 patterns - Reactive and unstable SMARTS filters. https://zinc20.docking.org/patterns/?reactive-gt=30.

[12] Mills JJ, Robinson KR, Zehnder TE, Pierce JG (2018). Synthesis and Biological Evaluation of the Antimicrobial Natural Product Lipoxazolidinone A. Angew. Chem. Int. Ed..

[13] Lu H (2023). Total Synthesis of the 2,5-Disubstituted γ-Pyrone E1 UAE Inhibitor Himeic Acid A. Org. Lett..

[14] Ponzo MG, Evindar G, Batey RA (2002). An efficient protocol for the formation of aminothiatriazoles from thiocarbamoylimidazolium salts. Tetrahedron Lett..

[15] Batey RA, Powell DA (2000). A General Synthetic Method for the Formation of Substituted 5-Aminotetrazoles from Thioureas:  A Strategy for Diversity Amplification. Org. Lett..

[16] Gavrilyuk JI, Evindar G, Chen JY, Batey RA (2007). Peptide-Heterocycle Hybrid Molecules:  Solid-Phase-Supported Synthesis of Substituted N-Terminal 5-Aminotetrazole Peptides via Electrocyclization of Peptidic Imidoylazides. J. Comb. Chem..

[17] Irwin JJ (2020). ZINC20—A Free Ultralarge-Scale Chemical Database for Ligand Discovery. J. Chem. Inf. Model..

[18] Kosowan JR, W’Giorgis Z, Grewal R, Wood TE (2015). Truce–Smiles rearrangement of substituted phenyl ethers. Org. Biomol. Chem..

[19] Henderson ARP, Kosowan JR, Wood TE (2017). The Truce–Smiles rearrangement and related reactions: a review. Can. J. Chem..

[20] Fuss D, Wu YQ, Grossi MR, Hollett JW, Wood TE (2018). Effect of the tether length upon Truce-Smiles rearrangement reactions. J. Phys. Org. Chem..

[21] Lofstrand VA, West FG (2016). Efficient Trapping of 1,2-Cyclohexadienes with 1,3-Dipoles. Chem. – Eur. J..

[22] Lofstrand VA, McIntosh KC, Almehmadi YA, West FG (2019). Strain-Activated Diels-Alder Trapping of 1,2-Cyclohexadienes: Intramolecular Capture by Pendent Furans. Org. Lett..

[23] Yamano MM (2019). Cycloadditions of Oxacyclic Allenes and a Catalytic Asymmetric Entryway to Enantioenriched Cyclic Allenes. Angew. Chem. Int. Ed..

[24] Jankovic, Christian L, West FG (2022). 2 + 2 Trapping of Acyloxy-1,2-cyclohexadienes with Styrenes and Electron-Deficient Olefins. Org. Lett..

[25] Smallworld and Arthor Databases - DISI. https://wiki.docking.org/index.php?title=Smallworld_and_Arthor_Databases.

[26] Landrum, G. *RDKit: A Software Suite for Cheminformatics, Computational Chemistry, and Predictive Modeling*. (Academic Press, 2013).

[27] Bickerton GR, Paolini GV, Besnard J, Muresan S, Hopkins AL (2012). Quantifying the chemical beauty of drugs. Nat. Chem..

[28] Baell JB, Holloway GA (2010). New Substructure Filters for Removal of Pan Assay Interference Compounds (PAINS) from Screening Libraries and for Their Exclusion in Bioassays. J. Med. Chem..

[29] Brenk R (2008). Lessons Learnt from Assembling Screening Libraries for Drug Discovery for Neglected Diseases. ChemMedChem.

[30] Doveston RG (2014). A unified lead-oriented synthesis of over fifty molecular scaffolds. Org. Biomol. Chem..

[31] Jadhav A (2010). Quantitative Analyses of Aggregation, Autofluorescence, and Reactivity Artifacts in a Screen for Inhibitors of a Thiol Protease. J. Med. Chem..

[32] Lipinski CA, Lombardo F, Dominy BW, Feeney PJ (2001). Experimental and computational approaches to estimate solubility and permeability in drug discovery and development settings1PII of original article: S0169-409X(96)00423-1. The article was originally published in Advanced Drug Delivery Reviews 23 (1997) 3–25.1. Adv. Drug Deliv. Rev..

[33] Veber DF (2002). Molecular Properties That Influence the Oral Bioavailability of Drug Candidates. J. Med. Chem..

[34] Bemis GW, Murcko MA (1996). The properties of known drugs. 1. Molecular frameworks. J. Med. Chem..

[35] Sauer WHB, Schwarz MK (2003). Molecular Shape Diversity of Combinatorial Libraries:  A Prerequisite for Broad Bioactivity. J. Chem. Inf. Comput. Sci..

[36] (2023). Zenodo.

[37] Bienfait B, Ertl P (2013). JSME: a free molecule editor in JavaScript. J. Cheminformatics.

[38] Chart.js - Open source JavaScript charting library. https://www.chartjs.org/.

[39] Bedart C (2024). Zenodo.

[40] Patel, H. *et al*. Synthetically Accessible Virtual Inventory (SAVI) Database - Building Blocks download. CADD Group, CBL, CCR, NCI, NIH 10.35115/37N9-5738 (2020).

[41] Hartung IV, Huck BR, Crespo A (2023). Rules were made to be broken. Nat. Rev. Chem..

[42] Hartenfeller M (2011). A collection of robust organic synthesis reactions for in silico molecule design. J. Chem. Inf. Model..

[43] Grygorenko OO (2020). Generating Multibillion Chemical Space of Readily Accessible Screening Compounds. iScience.

[44] Kondratov IS, Moroz YS, Grygorenko OO, Tolmachev AA (2022). The Ukrainian Factor in Early-Stage Drug Discovery in the Context of Russian Invasion: The Case of Enamine Ltd. ACS Med. Chem. Lett..

[45] Zhou JZ, Shi S, Na J, Peng Z, Thacher T (2009). Combinatorial library-based design with Basis Products. J. Comput. Aided Mol. Des..

[46] Sadybekov AA (2022). Synthon-based ligand discovery in virtual libraries of over 11 billion compounds. Nature.

[47] Ackloo S (2022). CACHE (Critical Assessment of Computational Hit-finding Experiments): A public–private partnership benchmarking initiative to enable the development of computational methods for hit-finding. Nat. Rev. Chem..

